# Blood Inflammatory-like and Lung Resident-like Eosinophils Affect Migration of Airway Smooth Muscle Cells and Their ECM-Related Proliferation in Asthma

**DOI:** 10.3390/ijms24043469

**Published:** 2023-02-09

**Authors:** Airidas Rimkunas, Andrius Januskevicius, Egle Vasyle, Jolita Palacionyte, Ieva Janulaityte, Skaidrius Miliauskas, Kestutis Malakauskas

**Affiliations:** 1 Laboratory of Pulmonology, Department of Pulmonology, Lithuanian University of Health Sciences, LT-44307 Kaunas, Lithuania; 2 Department of Pulmonology, Lithuanian University of Health Sciences, LT-44307 Kaunas, Lithuania; 3 Department of Laboratory Medicine, Lithuanian University of Health Sciences, LT-44307 Kaunas, Lithuania

**Keywords:** extracellular matrix, asthma, airway remodeling, migration, proliferation, adhesion, eosinophil subtypes, airway smooth muscle

## Abstract

Airway remodeling is a hallmark feature of asthma, and one of its key structural changes is increased airway smooth muscle (ASM) mass and disturbed extracellular matrix (ECM) homeostasis. Eosinophil functions in asthma are broadly defined; however, we lack knowledge about eosinophil subtypes’ interaction with lung structural cells and their effect on the airway’s local microenvironment. Therefore, we investigated the effect of blood inflammatory-like eosinophils (iEOS-like) and lung resident-like eosinophils (rEOS-like) on ASM cells via impact on their migration and ECM-related proliferation in asthma. A total of 17 non-severe steroid-free allergic asthma (AA), 15 severe eosinophilic asthma (SEA) patients, and 12 healthy control subjects (HS) were involved in this study. Peripheral blood eosinophils were enriched using Ficoll gradient centrifugation and magnetic separation, subtyped by using magnetic separation against CD62L. ASM cell proliferation was assessed by AlamarBlue assay, migration by wound healing assay, and gene expression by qRT-PCR analysis. We found that blood iEOS-like and rEOS-like cells from AA and SEA patients’ upregulated genes expression of contractile apparatus proteins, COL1A1, FN, TGF-β1 in ASM cells (*p* < 0.05), and SEA eosinophil subtypes demonstrated the highest effect on sm-MHC, SM22, and COL1A1 gene expression. Moreover, AA and SEA patients’ blood eosinophil subtypes promoted migration of ASM cells and their ECM-related proliferation, compared with HS (*p* < 0.05) with the higher effect of rEOS-like cells. In conclusion, blood eosinophil subtypes may contribute to airway remodeling by upregulating contractile apparatus and ECM component production in ASM cells, further promoting their migration and ECM-related proliferation, with a stronger effect of rEOS-like cells and in SEA.

## 1. Introduction

Chronic eosinophilic airway inflammation is an important feature seen in asthma, which leads to airway hyperreactivity and structural airway changes, also described as a remodeling [1]. Airway remodeling involves structural epithelial changes, subepithelial fibrosis, and increased bronchial smooth muscle mass due to cell hyperplasia and hypertrophy [2]. However, recent studies suggest that one more significant airway remodeling cause could be the changes in extracellular matrix (ECM) homeostasis induced by pulmonary structural cell ECM components production and disturbed ECM protein decomposition enzyme (metalloproteinases (MMPs)) function [3,4]. ECM is a non-cellular structure of every tissue, comprising a diverse group of proteoglycans, glycosaminoglycans, proteins, and glycoproteins [5]. Various ECM structural and functional molecules create a microenvironment in which adjacent cells may communicate via cell–cell or cell–ECM interactions. The molecular ECM composition, amount, and structure can influence cell behavior, migration, differentiation, proliferation, viability, and polarity [6]. Although ECM is a key part of the cell microenvironment, abnormal accumulation of it due to chronic airway inflammation in asthma may lead to changes in tissue function and structure, thus contributing to the airway remodeling [7].

Eosinophilic asthma is defined as a distinct asthma phenotype most commonly found in adults and associated with blood, sputum, and tissue eosinophilia, basement membrane thickening, and responsiveness to corticosteroid treatment [8]. Furthermore, patients with severe eosinophilic asthma often display poor symptom control and impaired lung function, and they are at a higher risk of disease exacerbations [9]. One of the causes of eosinophilic asthma complexity might reside in the existence of recently revealed two distinct eosinophil subtypes that differ according to their role in asthma pathogenesis [10]. It was shown that under homeostatic conditions, there is a separate type of homeostatic or resident eosinophils in various organs, including the lungs [11], with different properties from inflammatory eosinophils that develop from the bone marrow and migrate directly to the sites of inflammation. In our previous studies, eosinophil subtypes, isolated from human peripheral blood, were confirmed as lung resident-like eosinophils (rEOS-like) and inflammatory-like eosinophils (iEOS-like) [12] by flow cytometer, and their biological properties were investigated, revealing that distinct eosinophil subtypes might have different importance in asthma pathogenesis.

During the airways’ reaction to an antigen, a cascade of processes directed by cytokine-producing T helper type 2 cells (Th2) or type 2 innate lymphoid cells (ILC2) starts [13], ultimately resulting in the attraction of eosinophils to the airway. After migrating to the lung through blood vessel endothelium, eosinophils adhere to pulmonary structural cells or ECM when their integrins recognize cell receptors or protein ligands [14]. Eosinophils are an important source of growth factors and cytokines, which could promote the production of ECM components by the pulmonary structural cells [15]. We hypothesized that eosinophil subtypes’ adhesion to airway smooth muscle (ASM) cells might differ from adhesion to ECM components. In addition, recent studies have shown that eosinophils can affect the pulmonary structural cell proliferation [16]; however, there is not enough data about eosinophil subtypes-disturbed ECM component production, which could also affect ASM cell behavior in an autocrine manner. Moreover, activated eosinophils may produce reactive oxygen species (ROS), which are known to cause oxidative stress in the allergic asthma [17]. To better understand the differences in eosinophil subtypes’ effect on lung structural cells’ physiological activity, we investigated eosinophil subtypes’ spontaneous ROS production.

Accumulation of lung structural cells is determined by their altered apoptosis, deposition of ECM components, and increased proliferation. In addition, rising evidence suggests ASM cell migration is an important contributing feature to excessive ASM mass in the asthmatic airways [18]; however, there is little known about specific external mediators and their signaling pathways involved in ASM cell migration, especially in the context of asthma pathogenesis. These processes are stimulated by various factors produced by airway epithelial cells, ASM cells, myofibroblasts, and inflammatory cells that migrate to the site of inflammation. While it is known that ASM cells exhibit cellular plasticity [19] and contribute to airway remodeling, the regulation of its contractile, synthetic, and proliferative properties during eosinophilic inflammation is not fully understood. Thus, we decided to investigate eosinophil subtypes’ effect on ASM cell migration, ECM-related proliferation, and main ECM-related COL1A1, FN, TGF-β1, and contractile apparatus protein gene expression in asthma.

## 2. Results

### 2.1. Study Subject Characteristics

This study included 17 non-severe steroid-free allergic asthma (AA) patients, 15 severe eosinophilic asthma (SEA) patients, and 12 healthy subjects (HS) as the control group. The demographic and clinical characteristics of the population of this study are shown in Table 1. All individuals diagnosed with AA were allergic to Dermatophagoides pteronyssinus (D. pteronyssinus) house dust mites. Peripheral blood eosinophils count, fractional exhaled nitric oxide (FeNO), and immunoglobulin E (IgE) in patients with AA and SEA were significantly increased compared to the HS group. In addition, the AA and SEA groups showed significantly worse lung function compared to the HS group. Comparing asthma phenotypes, SEA patients had lower FEV_1_ and increased absolute blood eosinophil count, while AA patients displayed higher IgE.

### 2.2. Eosinophil Subtypes Adhesion to ASM Cells or Their Secreted ECM Components

Previously, we demonstrated that distinct eosinophil subtypes from the blood of asthma patients are characterized by enhanced adhesion to ASM cells [12]. During the current study, we investigated the differences between eosinophil subtypes’ attachment to ASM cells and their secreted ECM components. After 1 h of incubation in the AA group, 82.1 ± 2.5% of iEOS-like cells and 87.7 ± 2.3% of rEOS-like cells were stably attached to ECM components vs. 60.0 ± 2.6% of iEOS-like (*p* < 0.001) and 68.9 ± 2.5% of rEOS-like (*p* < 0.001) cells to ASM cells. In the SEA group, 84.3 ± 2.1% of iEOS-like and 91.2 ± 1.8% of rEOS-like cells stably adhered to ECM components, and 53.7 ± 2.5% of iEOS-like (*p* < 0.001) and 62.9 ± 2.7% of rEOS-like (*p* < 0.001) cells adhered to ASM cells. In the HS group, 70.4 ± 2.8% of iEOS-like cells and 75.5 ± 3.0% of rEOS-like cells were stably attached to ECM components and 43.2 ± 3.8% of iEOS-like (*p* < 0.01) and 50.4 ± 4.3% of rEOS-like (*p* < 0.01) cells were attached to ASM cells.

**Figure 1:**
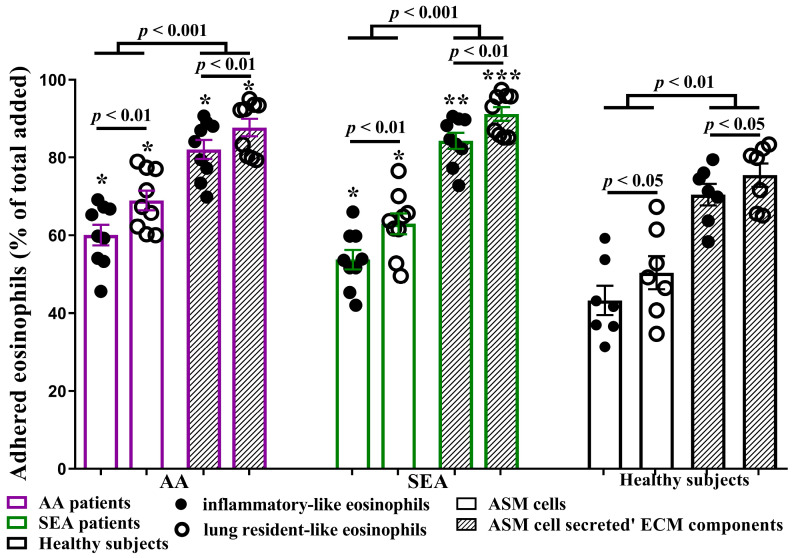
Eosinophil subtypes adhesion to ASM cells or their secreted ECM components. ASM—airway smooth muscle. ECM—extracellular matrix. AA (allergic asthma) n = 9. SEA (severe eosinophilic asthma) n = 9. Healthy subjects n = 7. Results are shown as mean ± SEM. * *p* < 0.05. ** *p* < 0.01. *** *p* < 0.001—compared with the respective eosinophil subtype of the healthy subject group. Statistical analysis: between investigated groups—Mann–Whitney two-sided U-test for independent data; within one study group—Wilcoxon matched-pairs signed-rank two-sided test for dependent data, comparing the iEOS-like and rEOS-like cells of each study participant separately. Lines denote comparison groups with a *p*-value indicating the significant difference in pairwise comparisons.

In all investigated groups, both eosinophil subtypes’ adhesion to ECM components was significantly higher than adhesion to ASM cells. There were no significant differences between asthma groups; however, eosinophil subtypes’ attachment to ASM cells or their secreted ECM components in asthma groups was significantly increased compared to the HS group. Furthermore, in all investigated groups, rEOS-like cells’ adhesion to ASM cells or their secreted ECM components was significantly higher (*p* < 0.05) than the iEOS-like cells (Figure 1).

### 2.3. Eosinophil Subtypes Effect on Gene Expression of TGF-β1, Primary Fibril and Contractile Apparatus Proteins in ASM Cells

Gene expression of primary fibril proteins collagen type I *α* 1 chain (COL1A1) and fibronectin (FN), as well as transforming growth factor (TGF)-*β*1, an important mediator involved in airway remodeling [20], were investigated in ASM cells after incubation with blood eosinophil subtypes. After 24 h of co-culture with iEOS-like and rEOS-like cells from AA and SEA patients, COL1A1, FN, and TGF-*β*1 gene expression in ASM cells significantly increased, compared with the HS group (*p* < 0.05) and the control ASM cells incubated without eosinophils (*p* < 0.01). No significant effect on COL1A1, FN, and TGF-*β*1 gene expression was detected in ASM cells after incubation with HS eosinophil subtypes. Furthermore, SEA patients’ rEOS-like cells’ effect was significantly higher than iEOS-like cells—COL1A1 expression was upregulated by 3.5 ± 0.2-fold vs. 2.7 ± 0.2-fold (*p* < 0.01); FN by 3.5 ± 0.4-fold vs. 2.3 ± 0.3-fold (*p* < 0.01); TGF-*β*1 by 2.7 ± 0.2-fold vs. 1.9 ± 0.2-fold (*p* < 0.01). This tendency in the AA group was only observed with FN—rEOS-like cells upregulated FN gene expression in ASM cells by 2.5 ± 0.2-fold, while iEOS-like cells by 1.8 ± 0.1-fold (*p* < 0.01).

**Figure 2:**
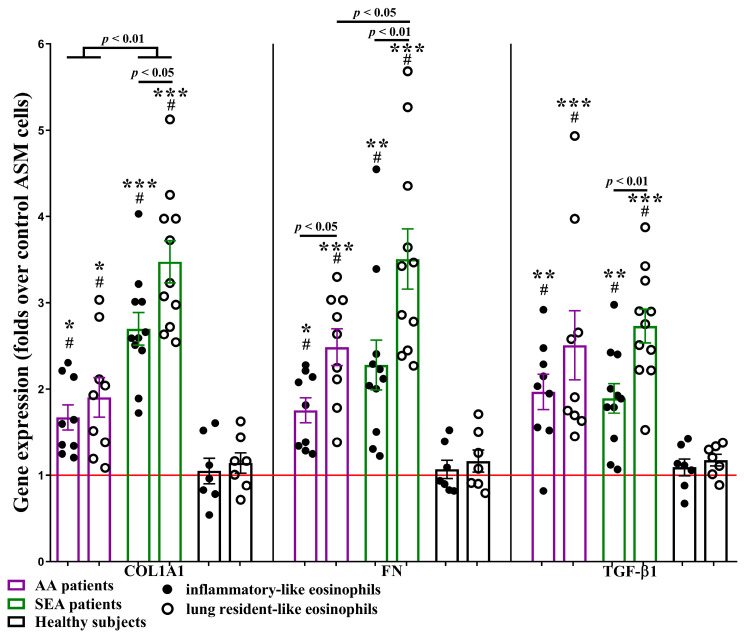
Gene expression in ASM cells after incubation with eosinophil subtypes. ASM—airway smooth muscle. COL1A1—collagen type I *α* 1 chain. FN—fibronectin. TGF-*β*1—transforming growth factor *β*1. AA (allergic asthma) n = 9. SEA (severe eosinophilic asthma) n = 11. Healthy subjects n = 7. Results are shown as mean ± SEM. # *p* < 0.01—compared with control ASM cells incubated without eosinophil subtypes. * *p* < 0.05. ** *p* < 0.01. *** *p* < 0.001—compared with the respective eosinophil subtype of the healthy subject group. Statistical analysis—between investigated groups—Mann–Whitney two-sided U-test for independent data. Within one study group—Wilcoxon matched-pairs signed-rank two-sided test for dependent data, comparing the iEOS-like and rEOS-like cells of each study participant separately. Lines denote comparison groups with a *p*-value indicating the significant difference in pairwise comparisons.

Moreover, blood eosinophil subtypes from SEA patients displayed an enhanced effect on COL1A1 gene expression in ASM cells, compared with AA group—2.7 ± 0.2-fold vs. 1.7 ± 0.2-fold (*p* < 0.01) for iEOS-like cells and 3.5 ± 0.2-fold vs. 1.9 ± 0.2-fold for rEOS-like cells (*p* < 0.01). Finally, SEA rEOS-like cells’ effect on FN gene expression in ASM cells was significantly higher than the AA patients’ rEOS-like cells effect (*p* < 0.05). No significant differences between asthma groups were noticed in the ASM cells’ TGF-*β*1 gene expression (Figure 2).

**Figure 3:**
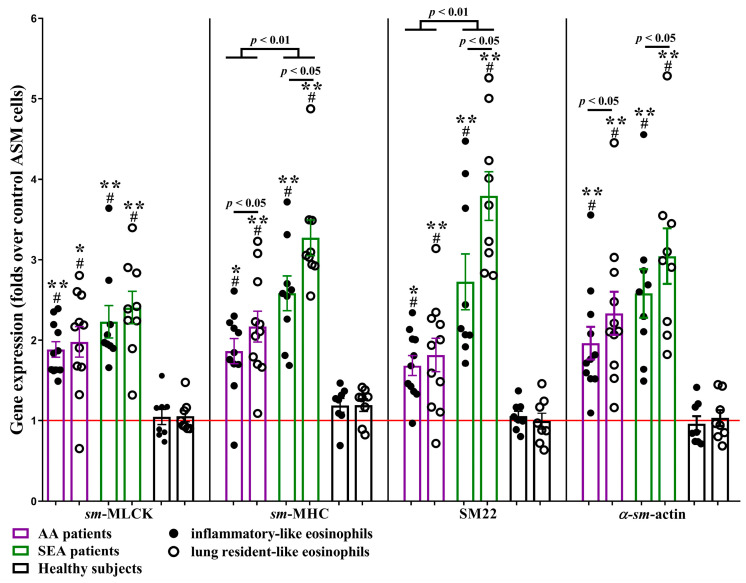
Gene expression in ASM cells after incubation with eosinophil subtypes. ASM—airway smooth muscle. *sm*-MLCK—smooth muscle myosin light chain kinase. *sm*-MHC—smooth muscle myosin heavy chain. SM22—transgelin. *α*-*sm*-actin—*α* smooth muscle actin. AA (allergic asthma) n = 11; SEA (severe eosinophilic asthma) n = 9. Healthy subjects n = 8. Results are shown as mean ± SEM. # *p* < 0.01—compared with control ASM cells incubated without eosinophil subtypes. * *p* < 0.01. ** *p* < 0.001—compared with the respective eosinophil subtype of the healthy subject group. Statistical analysis—between investigated groups—Mann–Whitney two-sided U-test for independent data; within one study group—Wilcoxon matched-pairs signed-rank two-sided test for dependent data, comparing the iEOS-like and rEOS-like cells of each study participant separately. Lines denote comparison groups with a *p*-value indicating the significant difference in pairwise comparisons.

In addition to investigating eosinophil subtypes’ effect on ASM cell migration, we evaluated several contractile apparatus protein gene expressions in ASM cells after incubation with eosinophil subtypes. Gene expression of contractile apparatus proteins -smooth muscle *(sm)*-myosin light chain kinase (MLCK), *sm*-myosin heavy chain (MHC), transgelin (SM22), and *α* smooth muscle actin (*α*-*sm*-actin) was upregulated in ASM cells after 24 h incubation with the AA and SEA patients’ eosinophil subtypes compared with HS group (*p* < 0.05) and control ASM cells, incubated without eosinophils (*p* < 0.01). In addition, SEA patients’ rEOS-like cells’ effect was significantly higher than iEOS-like cells—*sm*-MHC expression was upregulated by 3.3 ± 0.2-fold vs. 2.6 ± 0.2-fold (*p* < 0.05), SM22 by 3.8 ± 0.3-fold vs. 2.7 ± 0.3-fold (*p* < 0.05), and *α*-*sm*-actin by 3.1 ± 0.4-fold vs. 2.6 ± 0.3-fold (*p* < 0.05). A similar tendency in the AA group was observed—rEOS-like cells upregulated *sm*-MHC gene expression by 2.2 ± 0.2-fold vs. 1.9 ± 0.2-fold (*p* < 0.05) compared to iEOS-like cells and *α*-*sm*-actin by 2.3 ± 0.3-fold vs. 2.0 ± 0.2-fold (*p* < 0.05). Lastly, eosinophil subtypes from SEA patients displayed a higher effect on *sm*-MHC and SM22 gene expression in the ASM cells (*p* < 0.05) compared with AA patients’ respective eosinophil subtypes (Figure 3).

### 2.4. Migration of ASM Cells after Incubation with Eosinophil Subtypes

**Figure 4:**
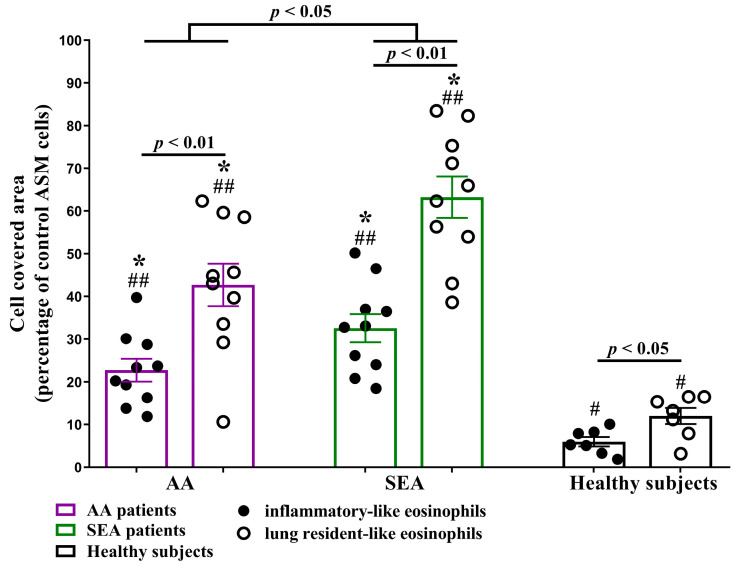
ASM cell migration after 72 h incubation with eosinophil subtypes. ASM—airway smooth muscle. AA (allergic asthma) n = 10, SEA (severe eosinophilic asthma) n = 10, healthy subjects n = 7. Results are shown as mean ± SEM. * *p* < 0.01—compared to the respective eosinophil subtype effect in the healthy subject group. # *p* < 0.05, ## *p* < 0.01—compared to control ASM cells. Statistical analysis—between investigated groups—Mann–Whitney two-sided U-test for independent data; within one study group—Wilcoxon matched-pairs signed-rank two-sided test for dependent data, comparing the iEOS-like and rEOS-like cells of each study participant separately. Lines denote comparison groups with a *p*-value indicating the significant difference in pairwise comparisons.

Previously, we demonstrated that ASM cell migration after incubation with asthmatic eosinophils significantly increased compared to healthy subjects [21]. In this study, we investigated distinct eosinophil subtypes’ effect on ASM cell migration. After 72 h of co-culturing, eosinophil subtypes from all investigated groups significantly promoted ASM cell migration (*p* < 0.05) compared to control ASM cells incubated without eosinophils. In addition, rEOS-like cells’ effect on ASM cell migration was significantly higher (*p* < 0.05) in all investigated groups compared to iEOS-like cells. ASM cell migration after incubation with AA and SEA patients eosinophil subtypes were significantly enhanced (*p* < 0.01), compared to the respective HS group eosinophil effect—in the AA group, ASM cells covered area was 22.7 ± 2.7 vs. 6.0 ± 1.1 percentage enhanced (expressed as a percentage of wounded and ASM cell-covered areas from control ASM cells after 72 h that were not incubated with eosinophil subtypes) after incubation with iEOS-like cells and 42.7 ± 5.0 vs. 12.0 ± 1.9 percentage of ASM cells after incubation with rEOS-like cells; in SEA group 32.6 ± 3.3 vs. 6.0 ± 1.1 and 63.3 ± 4.9 vs. 12.0 ± 1.9 percentage of ASM cells after incubation with iEOS-like and rEOS-like cells, respectively (expressed as a percentage of wounded and ASM cell-covered areas from control ASM cells after 72 h that were not incubated with eosinophil subtypes). Finally, SEA patients’ eosinophil subtypes’ effect on ASM cell migration was significantly higher (*p* < 0.05) compared to AA patients’ respective eosinophil subtypes’ effect (Figure 4).

### 2.5. ASM Cell ECM-Related Proliferation after Incubation with Eosinophil Subtypes

Considering that the response of lung structural cells to changes in their surrounding ECM components may contribute to airway remodeling in asthma, we aimed to investigate the potential effect of blood eosinophil subtypes on ASM cell proliferation via self-produced ECM. We found that ECM components, isolated 48 h after ASM cell co-culturing with eosinophil subtypes, promoted newly seeded ASM cell proliferation in the AA group by 6.2 ± 0.7% and 12.9 ± 1.6% after incubation with iEOS-like and rEOS-like cells, respectively, compared to control ASM cells grown on ECM components which were produced by culturing ASM cells without eosinophils (*p* < 0.01).

**Figure 5:**
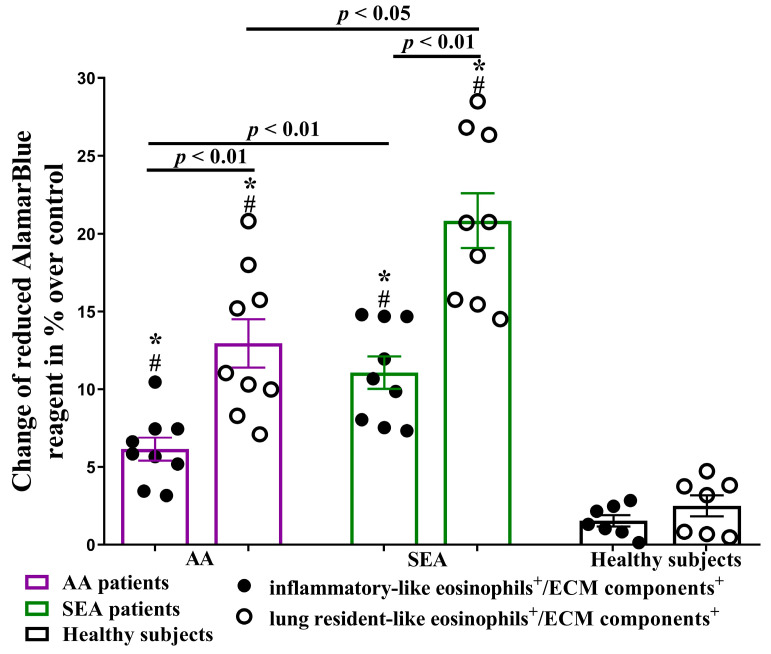
Newly seeded ASM cell proliferation on ECM components, purified after co-culturing ASM cells with eosinophil subtypes. ASM—airway smooth muscle. ECM—extracellular matrix. AA (allergic asthma) n = 9. SEA (severe eosinophilic asthma) n = 9. Healthy subjects n = 7. Results are shown as mean ± SEM. # *p* < 0.01—compared with control newly seeded ASM cell proliferation on ECM components, purified from ASM cell cultures without eosinophils. * *p* < 0.01—compared with the respective eosinophil subtype of the healthy subject group. Statistical analysis—between investigated groups—Mann–Whitney two-sided U-test for independent data; within one study group—Wilcoxon matched-pairs signed-rank two-sided test for dependent data, comparing the iEOS-like and rEOS-like cells of each study participant separately. Lines denote comparison groups with a *p*-value indicating the significant difference in pairwise comparisons.

Similarly, in the SEA group, ASM cell proliferation was increased by 11.1 ± 1.0% and 20.8 ± 1.8% after incubation with iEOS-like and rEOS-like cells, respectively (*p* < 0.01), compared to control ASM cell proliferation. In the HS group, no significant ASM cell proliferation was observed. In the AA and SEA groups, the rEOS-like cells’ effect was significantly higher compared with the iEOS-like cells’ (*p* < 0.01) effect, and both asthma groups patients’ eosinophil subtypes’ effect was higher compared to the HS group (*p* < 0.01). Furthermore, ECM components isolated after ASM cell co-culturing with SEA group eosinophil subtypes significantly enhanced (*p* < 0.05) newly seeded ASM cell proliferation compared with AA group (Figure 5).

### 2.6. Eosinophil Subtypes Spontaneous ROS Production

In bronchial asthma, eosinophils could be activated by various mediators, increasing their spontaneous ROS production, thus aggravating airway inflammation in the asthma [22,23]. Moreover, it could induce surrounding cells’ apoptosis and necrosis [24], affecting eosinophils’ proliferative properties. For this reason, we decided to investigate blood eosinophil subtypes’ spontaneous ROS production in asthma. We found that in the AA group, iEOS-like cells’ spontaneous ROS production was significantly higher than the production of the rEOS-like cells—111 ± 12.9 MFI vs. 62.1 ± 7.9 MFI (*p* < 0.01).

**Figure 6:**
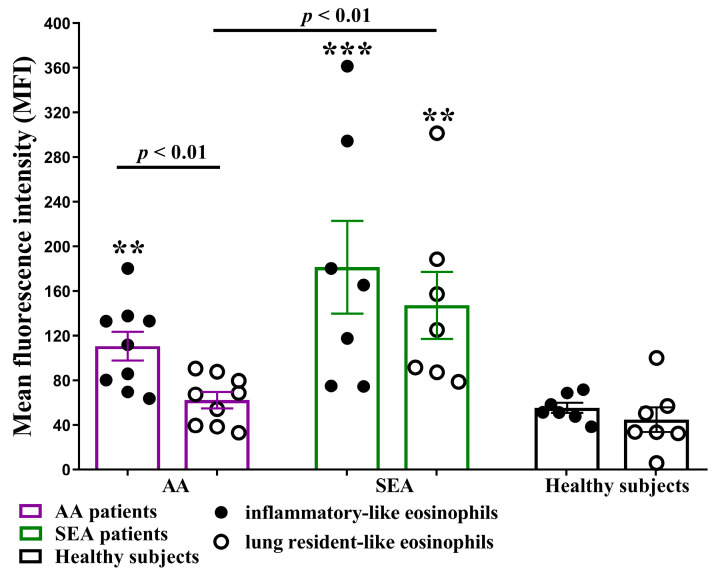
Spontaneous ROS production in blood eosinophil subtypes. AA (allergic asthma) n = 9. SEA (severe eosinophilic asthma) n = 7. Healthy subjects n = 7. Results are shown as mean ± SEM. ** *p* < 0.01. *** *p* < 0.001—compared with the respective eosinophil subtype of the healthy subject group. Statistical analysis—between investigated groups—Mann–Whitney two-sided U-test for independent data; within one study group—Wilcoxon matched-pairs signed-rank two-sided test for dependent data, comparing the iEOS-like and rEOS-like cells of each study participant separately. Lines denote comparison groups with a *p*-value indicating the significant difference in pairwise comparisons.

No significant differences between eosinophil subtypes were found in the SEA and HS groups. However, SEA patients’ rEOS-like cells ROS production was significantly enhanced compared with the AA group—147 ± 29.9 MFI vs. 62.1 ± 7.9 MFI (*p* < 0.01). In both asthma groups, iEOS-like cells’ ROS production was significantly increased compared with the HS group, but only SEA patients’ rEOS-like cells displayed the same tendency (Figure 6).

## 3. Discussion

This study showed that blood iEOS-like and rEOS-like cells’ attachment to ECM components in all investigated groups is significantly enhanced compared with attachment to ASM cells, and this adhesion further increases in asthma. In AA and SEA, both eosinophil subtypes display increased adhesive properties, promote ASM cell migration and ECM-related proliferation, and upregulate contractile markers—*sm*-MLCK, *sm*-MHC, *α*-*sm*-actin, SM22—and COL1A1, FN, TGF-*β*1 gene expression in ASM cells, compared with HS eosinophil subtype effect. The rEOS-like cells displayed stronger adhesive properties and increased ASM cell migration more than iEOS-like cells in all investigated groups, while AA and SEA patients’ rEOS-like cells displayed a stronger effect on ECM-related proliferation and upregulated FN, *sm*-MHC, and *α*-*sm*-actin gene expressions in ASM cells. In contrast, AA patients’ blood iEOS-like cells’ spontaneous ROS production is significantly higher than rEOS-like cells, with a similar tendency in the SEA group. In addition, the *sm*-MHC, SM22, and COL1A1 gene expressions in ASM cells, ECM-related proliferation, and ASM cell migration after incubation with SEA patients’ eosinophil subtypes were significantly higher than respective AA patients’ eosinophil subtypes.

Eosinophils express seven integrins [25], which can mediate their bond to adhesion molecules, such as vascular cell adhesion molecule (VCAM)-1 and intercellular adhesion molecule (ICAM)-1, but also to ECM proteins laminin through *α*6*β*1, vitronectin through *α*M*β*2, fibronectin through *α*4*β*7 and *α*4*β*1 [26]. Enhanced eosinophil attachment in asthma may be explained by an increase in the expression of surface membrane integrins or a different state of the integrin activation [25]. ECM components provide more attachment sites for eosinophil integrins, which results in quicker eosinophil attachment to ECM component ligands than to ASM cells‘ adhesion molecules. During this study, it was also found that eosinophil subtypes, particularly rEOS-like cells, adhere more intensively to ASM cells’ secreted ECM components, possibly due to stable adhesion being required for their function in the tissue. Furthermore, in response to various bronchial epithelial stimuli (atmospheric pollutants, allergens, infectious agents), the airway epithelium is considered to produce and release three distinct cytokines, IL-33, IL-25, and thymic stromal lymphopoietin (TSLP) (together known as alarmins), thus stimulating eosinophils maturation, differentiation, and migration into the airways [27]. The data in this study suggests that among eosinophil subtypes, rEOS-like cells bind more actively to ECM components than to ASM cells, potentially due to the expression of more ECM component-specific integrins and adhesion molecules on the rEOS-like cells’ membrane surface. Another reason may be the existence of more particular ECM component integrins in the active state than active integrins specific for ASM cells, and these processes are further elevated in the asthma [28]. A mechanism explaining this could be that eosinophil recruitment from the bloodstream into the airways relies on blood eosinophils becoming activated, which leads to their attachment to the endothelium, extravasation, and further migration through the bronchial tissue by surface adhesion molecule interaction with the ECM. Therefore, eosinophil subtypes, particularly rEOS-like cells, could be more adjusted to interact with ECM components, especially in chronic inflammation, due to constant interaction with released mediators. Eosinophils, in their active state, may secrete proinflammatory mediators, such as granule proteins, cytokines, chemokines, leukotrienes, and MMPs [29]. It has been shown that suppression of eosinophil attachment to ASM cells using specific antibodies against eosinophil adhesion receptors was associated with suppression of the ASM cell proliferation [16]. However, the ability of eosinophils to attach to ASM cells secreted’ ECM has not been assessed, which means eosinophils could degranulate to release the same mediators into the microenvironment and act on adjacent cells. Based on the results of this study, new ways could be found in the future to block certain eosinophil surface integrins responsible for attachment to the ECM and to reduce the accumulation of eosinophils in the airways, thus inhibiting the development of airway remodeling during asthma.

ASM cell migration has been implicated as a significant contributing factor to excessive ASM mass in asthmatic airways [18]. Cellular migration and contractility are closely connected processes. For cell migration to happen, several steps of cell polarization, projection, attachment, traction, and contraction must occur synchronically to ensure the migration [18,30]. Wound healing is an essential biological response to injury, requiring collective cell migration and proliferation to close the wound [31]. In response to injury, the mechanism by which cells close wounds (migration or proliferation) may differ among cell types [32], and understanding these mechanisms is important in therapeutic clinical treatment and lung remodeling assessment. Freshly separated ASM cells are considered contractile; however, upon being cultured in serum-rich conditions, they can modulate from a “contractile” phenotype to a “proliferative-synthetic” phenotype that responds poorly to contractile agonists and possesses decreased expression of contractile proteins, such as *α-sm-*actin, *sm*-MLCK, and *sm*-MHC [33,34]. The proliferative-synthetic ASM cells are characterized by increased proliferative potential and the expression of CCL11 (eotaxin-1), CXC10 (interferon γ-induced protein 10), connective tissue growth factor (CTGF), FN, and COL1A1. In contrast, the contractile phenotype has upregulated expression of *α*-actin, MLCK, MHC, and SM22 [33]. During our study, we found that the gene expression of contractile markers—*α*-*sm*-actin, *sm*-MHC, SM22, *sm*-MLCK—and proliferative markers COL1A1 and FN increased in ASM cells after incubation with AA and SEA patients’ eosinophil subtypes, revealing that eosinophils promote ASM cells differentiation, which demonstrates the presence of both proliferative–synthetic and contractile ASM cells phenotypes. Furthermore, rEOS-like cells, historically deemed tissue homeostasis-regulating cells, seem to have an enhanced effect on ASM cells, indicating that their tissue-regulating functions are overexpressed. It is still unknown at what ratios ASM cell phenotypes coexist during in vivo and in vitro conditions [34] and whether the ratio of contractile vs. proliferative–synthetic cells contributes to functional tissue abnormalities. This is a relevant question for future research, which should aim to understand lung structural cell behavior and homeostatic eosinophil’s role in asthma pathogenesis better. An aberrant accumulation of lung structural cells and ECM proteins has been an acknowledged feature of asthma. Previously it has been demonstrated that ECM protein collagen I, III, V, fibronectin, and laminin, but not elastin, support ASM cell migration [35]. After migrating into the airways, eosinophils may produce various cytokines and chemokines, which could cause ASM cell activation and further stimulate them to produce various mediators [36], thus promoting autocrine ASM cell activation, resulting in increased migration and proliferation. In this study, asthma patients’ eosinophil subtypes, especially SEA patients’ rEOS-like cells, upregulated COL1A1, and FN gene expression in ASM cells, which could cause increased production of ECM proteins collagen I and fibronectin and subsequently promote ASM cell migration.

TGF-*β* plays diverse roles in mediating airway structural cell proliferation, differentiation, and contractile protein expressions by exerting its effect through its capacity to adjust the deposition of ECM components [37]. In various airway diseases, these lung structural cell function aspects play a fundamental role in disease pathology. It has been discussed that TGF-*β* expression can be upregulated in lung structural cells in airway disease and also increased in the airway after immune cell activation [38], with eosinophils potentially being the largest source of TGF-*β1* [39]. Furthermore, it has been shown to have an additive and/or synergistic effect with platelet-derived growth factor (PDGF) and facilitate ASM cell migration by modifying MMPs and tissue inhibitors of metalloproteinases balance through the extracellular signal-regulated kinase (ERK) pathway [40], as well as increase *α*-*sm*-actin, fibronectin, and collagen Ⅰ protein levels [41]. In this study, AA and SEA patients’ eosinophil subtypes upregulated TGF-*β*1 gene expression in ASM cells. However, only in the SEA group rEOS-like cells revealed a significantly enhanced effect compared to iEOS-like cells. It is known that TGF-*β* is secreted as the latent complex that accumulates in ECM and requires activation to be a functionally active molecule [42]. Several factors, such as ROS, proteases MMP-2 and MMP-9, pH, and integrins, contribute to the liberation of active TGF-*β* from ECM. Activated eosinophils can be a source of latent TGF-*β* liberating MMP-9 [43] and ROS [22], but also TGF-*β*1 [20] itself.

Airway remodeling is a hallmark feature of asthma which includes hyperplasia and hypertrophy of the ASM [1], and eosinophils potentially contributing to it [16]; however, not enough research has been made to evaluate the effects of ECM components on these processes. Based on the information previously provided, it appears that eosinophils can promote pro-proliferative ECM protein production, and these changes in the modified matrix could be able to feedback signals to the ASM cells in an autocrine manner within their environment to influence various cellular functions. One possible mechanism could be eosinophils promoting enhanced production of pro-proliferative ECM components, such as fibronectin and collagen I [44], by ASM cells. Lung structural cells naturally secrete ECM to maintain their microenvironment. However, during asthma, chronic inflammation promotes eosinophil migration, accumulation, and degranulation in the airways, which leads to subsequent excess ASM proliferation and dysregulation of ECM proteases and their inhibitors, resulting in ECM component deposition and accumulation in the airways.

Cell proliferation is associated with a diverse set of signaling pathways, which control cell differentiation, quiescence, senescence, and responses to various stress factors [45]. A proposed main mechanism for increased ASM mass is ASM cell proliferation, induced by various cytokines, growth factors, inflammatory mediators, and allergens [46]. In addition, emerging evidence has implicated enhanced ASM cell migration as an important contributing feature to excessive ASM mass in asthmatic airways [18]. Fully differentiated ASM cells can produce various cytokines and growth factors into the microenvironment of the airways [36,47] and further promote nearby cell proliferation and migration in a paracrine or autocrine manner. The rEOS-like cells or homeostatic eosinophils are considered “positive” because they express several genes related to tissue homeostasis and immune response regulation [10]. As a result of chronic inflammation in asthma, rEOS-like cells could be potentially overstimulated by various mediators, thus explaining their enhanced effect on ASM cell physiological activity. Meanwhile, iEOS-like cells can be considered eosinophils with a “negative” role due to their high expression of inflammatory genes. Due to the increased exposure to various mediators, iEOS-like cells, which first migrate to the epithelial area, are further activated, which may cause them to produce more ROS and have harmful effects on nearby lung structural cell homeostasis and functions. Our study revealed that iEOS-like cells’ effect on ASM cell migration and ECM-related proliferation is lesser than rEOS-like cells, a likely result of iEOS-like cells’ ability to produce higher amounts of spontaneous ROS, promoting ASM cell apoptosis and necrosis [48] rather than proliferation.

In contrast, our study had several limitations. Eosinophil subtypes were isolated using magnetic separation, magnetically marking rEOS-like cells as L-selectin (CD62L) positive and iEOS-like cells as L-selectin negative. However, we cannot claim that the iEOS-like cell population is pure due to other L-selectin negative blood cells contaminating the population. In addition, decellularization procedures have become favored substitutes to experimental plate coating with ECM components because the decellularization method aims to reduce any adverse effect on the arrangement, biological activity, and mechanical disorder of the remaining ECM after efficiently eliminating all cellular and nuclear materials [49,50]. In our study, we adapted an in vitro decellularization technique to mimic natural ECM assembly processes by enabling investigated hTERT human ASM cells to proliferate and produce their own ECM components rather than using commercial ECM products or pre-coated tissue culture plates for our experiments. The ECM component matrices obtained during the experiments maintain crucial structural components but may lack the related modulatory proteins or soluble components required to promote tissue-specific cellular functions. However, freshly seeded cells partially regenerate missing ECM components [51]. Moreover, the NH_4_OH used to remove the cell portion in this study may have cytotoxic effects on newly seeded cells. Still, this effect was minimized by optimizing the ECM washing protocol and further incubating tissue culture wells in PBS before seeding new ASM cells to remove NH_4_OH residue. Additionally, to minimize the influence of ASM cell proliferation in wound healing assay experiments, serum-free conditions were maintained. Furthermore, the SEA patients in this study were significantly older than AA patients and HS. It has been previously indicated that eosinophil degranulation, but not adhesive and chemotactic properties, was decreased in older asthma patients compared to younger patients [52]. Nevertheless, we state that the age difference does not bias our data as the research depended on the severity of the disease but not on the age groups. Lastly, a possible limitation of our gene expression experiments was that changes at the protein level were not evaluated. It is claimed that the number of transcripts may not always correlate with the protein level. However, differentially expressed messenger ribonucleic acids (mRNA) correlate significantly better with their protein product than non-differentially expressed mRNAs [53].

In conclusion, blood eosinophil subtypes, particularly rEOS-like cells, adhere more intensively to ECM components than to ASM cells, and this adhesion further increases in asthma. Furthermore, asthmatic patients’ blood rEOS-like and iEOS-like cells change ASM cells activity by upregulating their contractile apparatus and ECM protein gene expression, thus promoting ASM cells migration and ECM-related proliferation, with a stronger effect of rEOS-like cells and in SEA. Our data could provide a better understanding of eosinophil subtypes’ contribution to airway remodeling in asthma, which may help future treatment decisions and further improve patient outcomes.

## 4. Materials and Methods

### 4.1. Ethics

The research protocol was confirmed by the Lithuanian University of Health Sciences Committee of Regional Biomedical Research Ethics (BE-2-58). This study was registered in the U.S. National Institutes of Health ClinicalTrials.gov trial registry with the identifier NCT04542902.

### 4.2. Study Population

This study included 44 adults. The participants in this study were adult women and men aged 18–75 who had signed written informed consent before enrolling in this study. Investigated participants were all patients from the Department of Pulmonology, Hospital of the Lithuanian University of Health Sciences.

### 4.3. Study Design and Experimental Plan

**Figure 7:**
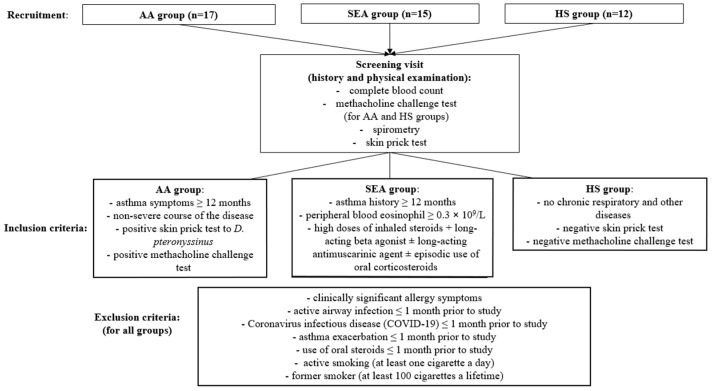
Inclusion and exclusion criteria for this study population. AA—allergic asthma. D. pteronyssinus—Dermatophagoides pteronyssinus. SEA—severe eosinophilic asthma.

To confirm the inclusion and exclusion criteria (Figure 7), all participants underwent physical examination, skin prick test, and spirometry. A methacholine challenge test was performed in AA and HS groups. If participants met the criteria, they were introduced to the requirements for participation in this study, and an informed written agreement was acquired.

**Figure 8:**
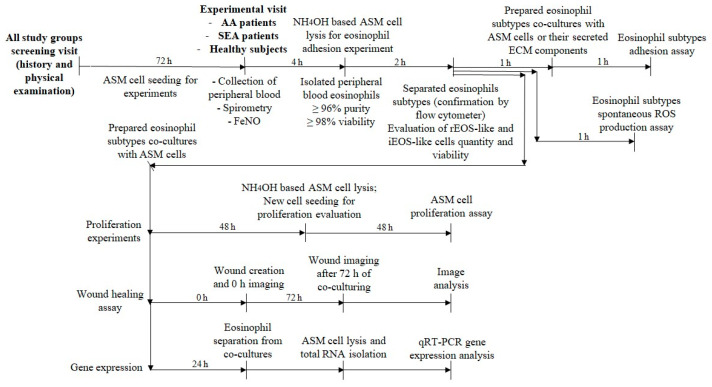
Study design and experimental plan. AA—allergic asthma. ASM—airway smooth muscle. Co-culture—combined cell cultures. ECM—extracellular matrix. FeNO—fractional exhaled nitric oxide. iEOS-like—inflammatory-like eosinophil. NH_4_OH—ammonium hydroxide. rEOS-like—lung resident-like eosinophil. RNA—ribonucleic acid. qRT-PCR—quantitative reverse transcriptase polymerase chain reaction. SEA—severe eosinophilic asthma.

Each investigated individual was examined according to the design of this study and experimental plan (Figure 8). ASM cells were seeded into cell culture plates 72 h before the patient’s experimental visit. During the experimental visit, peripheral blood was collected, and FeNO was evaluated. Separated blood eosinophils’ viability and the amount were assessed. Eosinophils were subtyped into iEOS-like cells and rEOS-like cells using magnetic separation, and their viability and amount were assessed. Then, eosinophil subtypes were used to create co-cultures with ASM cells or their secreted ECM components for eosinophil adhesion, ASM cell migration, gene expression, and proliferation experiments. If the eosinophils’ purity was <96% and viability <98%, the investigated subject was excluded from this study. All data presented in the manuscript were from the investigated subjects who passed these criteria.

### 4.4. Complete Blood Count and Immunoglobulin E

For each routine clinical chemistry test, investigated subjects’ blood samples were transported directly to our hospital laboratory. A complete blood count test was performed with an automated hematology analyzer UniCel^®^ DxH 800 Coulter^®^ Cellular Analysis System (Beckman Coulter, Miami, FL, USA), and immunoglobulin E (IgE) level was measured by automated immunoassay analyzer AIA-2000 (Tosoh Bioscience, South San Francisco, CA, USA).

### 4.5. Lung Function Testing

An ultrasonic spirometer (Ganshorn Medizin Electronic, Niederlauer, Germany) was used to examine lung function. The results of forced expiratory volume in 1 s (FEV_1_), forced vital capacity (FVC), and the FEV_1_/FVC ratio were considered the largest of the three independent measurements, as described in [12].

### 4.6. Methacholine Challenge Test

A methacholine challenge test was administered using a pressure dosimeter (ProvoX, Ganshorn Medizin Electronic, Niederlauer, Germany) to detect airway hyperresponsiveness. Investigated subjects were asked to inhale aerosolized methacholine at 2 min intervals until the total cumulative dose or a 20% decline in FEV_1_ from the baseline was reached. The bronchoconstriction effect of each methacholine dose was expressed as described in [12].

### 4.7. Skin Prick Testing

All examined individuals underwent skin prick allergy testing with standardized allergen extracts (Stallergenes, S.A., Antony, France) for D. Pteronyssinus and other clinically significant allergens. A 1% Histamine dihydrochloride solution (10 mg/mL) was applied as a positive control, and diluent (saline) as a negative control. The skin prick test was interpreted after 15 min of application. Skin prick test results were deemed positive if the mean wheal diameter was greater than 3 mm.

### 4.8. Fractional Exhaled Nitric Oxide Measurement

All examined individuals’ FeNO was measured by a handheld Vivatmo-me device (Bosch Healthcare Solutions, Waiblingen, Germany). Following a short self-calibration by the device, the examined individual was requested to exhale through the single-use mouthpiece in a steady flow for approximately 5 s. To minimize possible cross-contamination risk, the investigating personnel were properly protected with full personal protective equipment, and the Vivatmo-me device was thoroughly sanitized between uses together with single-use mouthpiece replacements.

### 4.9. ASM Cell Cultivation In Vitro

For every experiment, healthy human ASM cells immortalized by stable expression of the human telomerase reverse transcriptase (hTERT) gene as described by Gosens R. et al. [54] were used. ASM cells were grown in standard cultivation conditions at 37 °C and 5% CO_2_ in the air with cell culture medium renewal every three days. To avoid diminishing cell activity and viability after repeated amounts of cell passaging, new cells of the same mainline were thawed each time after 6 passages. ASM cells were cultivated in “Dulbecco’s modified Eagle’s medium” (DMEM) (GIBCO by Life Technologies, Paisley, UK), supplemented with cell growth supplement fetal bovine serum (FBS) (10% *v*/*v*; GIBCO), antibiotics penicillin and streptomycin (2% *v*/*v*; GIBCO) and antifungal amphotericin B (1% *v*/*v*; GIBCO). Before each experiment, ASM cells were serum-starved in DMEM, supplemented with penicillin/streptomycin, amphotericin B, insulin, transferrin, and selenium reagent (1% *v*/*v*, GIBCO) to stop following cell proliferation and reduce probable errors due to the effect of serum mediators. During experiments with eosinophil subtypes, control ASM cells were cultivated alongside co-cultures and used as a baseline point to evaluate the eosinophil subtype effect.

### 4.10. Blood Eosinophil Isolation, Purification, and Eosinophil Subtyping

Peripheral blood samples (approximately 30 mL) from each studied individual were collected into sterile vacutainers with dipotassium ethylenediaminetetraacetic acid (K_2_EDTA) (BD Biosciences, San Jose, CA, USA). Eosinophil isolation, purification, and subtyping were performed as previously described [12], using magnetic beads conjugated with antibodies against CD62L (Miltenyi Biotec, Bergisch Gladbach, Germany), which is expressed on rEOS-like cells, but not on iEOS-like cells [10]. Once separated, both cell populations were confirmed by flow cytometer FacsCalibur (BD Biosciences, San Jose, CA, USA), the total amount was counted, and cell viability was assessed by ADAM (NanoEnTek Inc, Mountain View, CA, USA).

### 4.11. ASM Cell-Secreted ECM Purification with NH_4_OH-Based Cell Lysis

**Figure 9:**
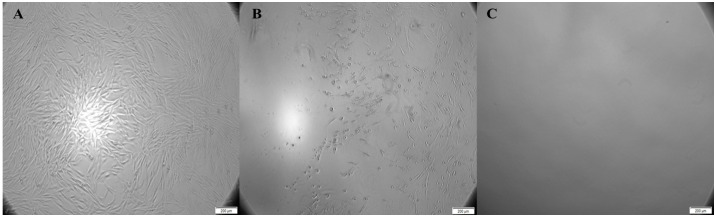
ASM cell culture visualization during NH_4_OH-based cell lysis. (**A**) ASM cells before NH_4_OH addition. (**B**) ASM cells after 3 min with NH_4_OH. (**C**) ASM cells after <10 min with NH_4_OH. Magnification in panels (**A**–**C**): wide-field eyepiece, ×10; objective, ×10.

ECM yield and purification protocols were adjusted to laboratory conditions [49,50]. To isolate the ASM cell-produced insoluble ECM layer, a fresh ammonium hydroxide (NH_4_OH) (Sigma-Aldrich Chemie GmbH, Taufkirchen, Germany) working solution for each patient’s visit was prepared and used to lyse the ASM cell cultures. NH_4_OH solution (50 mM) was added to each experimental well and incubated for up to 10 min. During the incubation, cells were monitored under an inverted light microscope (CETI, Inverso TC100, Medline Scientific, Chalgrove, UK) with an installed XM-10-IR-2 camera (Olympus, Tokyo, Japan) while constantly gently moving the tissue culture plate to lyse all cells completely, as shown in Figure 9. After observing no visible cells, the liquid was removed. Each well was carefully washed three times with PBS (GIBCO by Life Technologies, Paisley, UK) and incubated for 15 min with PBS to collect further and remove any remaining NH_4_OH residue.

For eosinophil adhesion experiments, after growing ASM cells for 72 h, the culture medium was removed, wells were washed once with PBS, and ECM components were purified as described earlier. Then, the ECM component layer at the bottom of the tissue culture plates was used for eosinophil subtypes attachment experiments.

For ASM cell proliferation on ECM experiments, after co-culturing ASM cells with eosinophil subtypes for 48 h, the medium was removed, and wells were rinsed with PBS. ECM was purified as described earlier. New ASM cells (1.5 × 10^4^ in each well) were seeded onto ECM-coated wells and incubated with DMEM (2% *v/v* FBS) for 48 h at standard conditions. After incubation, newly seeded ASM cell proliferation was evaluated using the AlamarBlue assay.

### 4.12. Eosinophil Subtypes Adhesion to ASM Cells or Their Secreted ECM Evaluation

ASM cells were seeded into 24-well tissue culture plates (CytoOne, StarLab, Brussels, Belgium) (1 × 10^4^ cells in each well) in DMEM, supplemented with FBS (10% *v/v*) and grown for 72 h at standard conditions. Afterward, the cell culture medium was removed, and every well was washed once with PBS. The culture medium was changed 24 h before the experiments by supplementing cells with a serum-free medium as described in Section 4.9. Wells dedicated to ECM experiments were lysed with NH_4_OH to extract ECM components, as shown in Section 4.11. After eosinophil enrichment and subtyping, co-cultures with ASM cells or their secreted ECM were prepared (1.25 × 10^4^ of an eosinophil subtype was added in each well). Eosinophil attachment to ASM cells or their secreted ECM components was measured after 1 h of co-culturing. After 1 h, the medium was aspirated, and wells were gently rinsed with PBS to collect any leftover non-attached eosinophils. Eosinophil adhesion was evaluated by measuring residual eosinophil peroxidase (EPO) activity. To evaluate EPO activity, 116 μL of EPO substrate (1 mM *o*-phenylenediamine, 1 mM H_2_O_2_, and 0.1% Triton X-100 in Tris buffer, pH 8.0) and 116 μL of DMEM medium without phenol red were added to each experimental well and incubated for 30 min at standard conditions. After the incubation, 58 μL of STOP reagent (sulfuric acid—H_2_SO_4_) was added to each investigated well to stop EPO activity, and the absorbance was measured at 490 nm wavelength by a microplate spectrophotometer. Results were represented as % of the attached eosinophil number from the total added, calculated from a control eosinophil subtype amount (1.25 × 10^4^ cells) absorbance result value.

### 4.13. RNA Isolation and Quantitative Reverse Transcriptase PCR Analysis

Before each experiment, ASM cells were seeded in 6-well cell culture plates (2 × 10^4^ cells in each well) and incubated as described in Section 4.9. After isolating blood eosinophil subtypes, co-cultures with ASM cells were prepared (5 × 10^4^ of an eosinophil subtype was added in each well). After 24 h incubation, eosinophil subtypes were removed from ASM cells for gene expression analysis. ASM cells were lysed using TRIzol™ reagent (Invitrogen™, UK), and total RNA was purified by utilizing miRNeasy Mini Kit (Qiagen, Maryland, USA) as stated by the manufacturer’s protocol. Quantitative reverse transcriptase polymerase chain reaction (qRT-PCR) analysis was accomplished by using an SYBR^®^ Green I based RNA-to-CT™ 1-Step Kit (Applied Biosystems, Waltham, Massachusetts, USA) in the Applied Biosystems 7500 Fast Real-Time PCR System according to the manufacturer’s instructions. Blood eosinophil subtypes’ effect on gene expression in ASM cells was evaluated as folds over the control ASM cells incubated without eosinophils. The 18S gene was used as a housekeeping gene. All primers used in ASM cell gene expression analysis are depicted in Table 2.

### 4.14. ASM Cell Migration—Wound Healing Assay

ASM cell migration was evaluated using an in vitro wound healing-scratch assay, as stated by Liang et al. [55]. Before each experiment, ASM cells were seeded in 6-well cell culture plates (2 × 10^4^ cells in each well) (CytoOne, StarLab, Brussels, Belgium) and incubated as described in Section 4.9. After isolating blood eosinophil subtypes, each well with ASM cells was scraped to imitate the cell migration during wound healing in vivo. After co-culturing ASM cells with eosinophils (5 × 10^4^ of an eosinophil subtype was added in each well), images were taken at the 0 h time point; then, after 72 h of incubation, a sufficient amount of time was needed to show differences in ASM cell migration [21]. All images were examined in ImageJ software (NIH and LOCI, University of Wisconsin), and the results were represented as a percentage of wounded and ASM cell-covered areas from control ASM cells that were not incubated with eosinophil subtypes.

### 4.15. AlamarBlue ASM Cell Proliferation Assay

For proliferation experiments, ASM cells were seeded in 24-well plates (2.5 × 10^3^ cells in each well) and incubated as described in Section 4.9. The cultivation medium was changed into the 2% FBS experiment medium before co-culturing with eosinophils. ASM cells were incubated with a group of isolated eosinophil subtypes (1.25 × 10^4^ of an eosinophil subtype was added in each well) from AA, SEA, or the HS group for 48 h. After incubation, cells were lysed with NH_4_OH to extract ECM components, as described in Section 4.11. Afterward, new ASM cells were seeded into each well in DMEM (2% *v*/*v* FBS) for 2 days. Following 48 h incubation, the culture medium was removed, and cell culture wells were washed with warm PBS. ASM cell proliferation on ECM components was assessed by adding Hank’s balanced salt and AlamarBlue reagent (10% *v*/*v*; Invitrogen™, UK). AlamarBlue conversion from blue-colored reagent resazurin to the reduced pink-colored form resofurin depended on the ASM cells’ metabolic activity, measured at wavelengths of 570 nm and 600 nm by a spectrophotometer. As indicated by the manufacturer, the portion of AlamarBlue conversion is directly proportional to the number of viable cells. The results were denoted as the percentage difference in AlamarBlue reagent conversion by ASM cells, grown on ECM components, produced by co-culturing ASM cells with eosinophil subtypes, compared with control ASM cells, grown on ECM components, and produced by ASM cells without co-culturing with eosinophils.

### 4.16. Evaluation of Spontaneous Reactive Oxygen Species Production in Eosinophil Subtypes

For each examined individual experiment, a fresh Dihydrorhodamine (DHR)-123 (Sigma-Aldrich, Chemie GmbH, Taufkirchen, Germany) dye working solution was prepared. DHR-123 is a fluorescence probe commonly used for measuring ROS [56]. It can passively diffuse through cell membranes and then, after being exposed to intracellular nitric oxide and peroxynitrite, DHR-123 is oxidized to rhodamine-123, which exhibits green fluorescence and stains mitochondria inside a living cell [57], thus allowing detection by flow cytometer. After isolating eosinophil subtypes, flow cytometer test tubes (Corning Falcon, Newport, Tennessee, USA) were prepared (5 × 10^4^ of an eosinophil subtype in each tube), and 1x PBS was added to a final volume of 200 μL. A separate test tube with 200 μL DHR-123 was prepared to evaluate reagent contamination. Each experimental tube was supplemented with DHR-123 working solution (final concentration 750 ng/mL), mixed by gentle vortexing, and incubated for 45 min, a sufficient amount of time for fluorescence evaluation [58], at standard conditions (5% CO_2_ in air at 37 °C). For flow cytometry calibration, a test tube with 5 × 10^4^ eosinophils in PBS, but without DHR-123, was prepared. After incubation, the relative amount of ROS formed was evaluated in a flow cytometer by measuring the mean fluorescence intensity (MFI) of the investigated eosinophil subtype population.

### 4.17. Statistical Analysis Methods

Statistical data analysis was conducted using GraphPad Prism 8 for Windows (ver. 8.0.1, 2018; GraphPad Software Inc., San Diego, CA, USA). Results are shown as mean ± standard error of the mean (SEM) unless mentioned otherwise. The Shapiro–Wilks normality test was implemented to examine the assumption of normality in data distribution. The data distribution did not pass the normality test; thus, non-parametric tests were used. Distinctions among two independent groups were evaluated using the Mann–Whitney two-sided U-test. The Wilcoxon matched-pairs signed-rank two-sided test was used for two dependent groups. A *p* < 0.05 value was considered statistically significant.

## Figures and Tables

**Table 1:** Demographic and clinical characteristics of study population.

	AA	SEA	HS
Number, n	17	15	12
Age, median (range), years	26 (18–53)	60 (45–75) **^†^	27 (23–53)
Sex (male/female), n	11/6	2/13	5/7
BMI, median (range) kg/m^2^	23.2 (17.5–34.1)	29.1 (17.6–39.8) *^#^	23.2 (19.3–33.1)
Sensitization to D. pteronyssinus (patients), n	17	2	0
FEV_1_, L	3.4 ± 0.2	1.4 ± 0.1 **^†^	4.0 ± 0.2
FEV_1_, % of predicted	83.5 ± 2.6 *	57.3 ± 4.6 **^†^	101.8 ± 2.9
PD_20_	0.17 ± 0.03	ND	NR
Blood eosinophil count (×10^9^)/L	0.37 ± 0.06 **	0.57 ± 0.09 **^#^	0.15 ± 0.02
Blood eosinophil count, %	5.9 ± 0.8 **	7.8 ± 1.1 **	2.5 ± 0.4
IgE, IU/mL	558.7 ± 213.0 **	132.5 ± 33.6 **^#^	21.7 ± 5.1
FeNO, ppb	34.8 ± 5.0 **	36.9 ± 5.5 **	12.0 ± 2.0

AA—allergic asthma. BMI—body mass index. D. pteronyssinus—Dermatophagoides pteronyssinus. FeNO—fractional exhaled nitric oxide. FEV_1_—Forced expiratory volume in 1 s. IgE—immunoglobulin E. HS—healthy subjects. ND—not done. NR—no response. SEA—severe eosinophilic asthma. PD20—provocation dose of methacholine resulting in a 20% diminish in FEV_1_. Data presented as mean ± standard error of mean. * *p* < 0.05. ** *p* < 0.01 compared to the healthy subject group. # *p* < 0.05, † *p* < 0.01 compared with the AA group. Statistical analysis—Mann–Whitney two-sided U-test between two independent groups.

**Table 2:** Primers used for gene expression analysis.

Gene	Forward 5′–3′	Reverse 5′–3′
18S	CGCCGCTAGAGGTGAAATTC	TTGGCAAATGCTTTCGCTC
*α*-*sm*-actin	TGGGTGACGAAGCAC AGAGC	CTTCAGGGGCAACACGAAGC
*sm*-MHC	CGCCAAGAGACTCGTCTGG	TCTTTCCCAACCGTGACCTTC
SM22	AGGAGCGGCTGGTGGAGTGGAT	CATGTCAGTCTTGATGACCCCATAGT
*sm*-MLCK	GACTGCAAGATTGAAGGATAC	GTTTCCACAATGAGCTCTGC
COL1A1	TCGAGGAGGAAATTCCAATG	ACACACGTGCACCTCATCAT
FN	AGCCAGCAGATCGAGAACAT	TCTTGTCCTTGGGGTTCTTG
TGF-*β*1	GTACCTGAACCCGTGTTGCT	GAACCCGTTGATGTCCACTT

18S—18S ribosomal RNA reference gene; *α*-*sm*-actin—*α* smooth muscle actin gene; *sm*-MHC—smooth muscle myosin heavy chain gene; SM22—transgelin gene; *sm*-MLCK—smooth muscle myosin light chain kinase gene; COL1A1—collagen type I *α* 1 Chain gene; FN—fibronectin gene; TGF-*β*1—transforming growth factor-*β*1 gene.

## Data Availability

All the data presented in this study are included in this article.

## Abbreviations

AA	allergic asthma
ASM	airway smooth muscle
Co-culture	combined cell culture
COL1A1	collagen type I *α* 1 chain
DHR	Dihydrorhodamine
D. pteronyssinus	Dermatophagoides pteronyssinus
DMEM	Dulbecco’s modified Eagle’s medium
ECM	extracellular matrix
EPO	eosinophil peroxidase
ERK	extracellular signal-regulated kinase
FBS	fetal bovine serum
FeNO	fractional exhaled nitric oxide
FEV_1_	forced expiratory volume in 1 s
FN	fibronectin
FVC	forced vital capacity
hTERT	human telomerase reverse transcriptase
HS	healthy subject
ICAM-1	intercellular adhesion molecule 1
iEOS	inflammatory eosinophil
IgE	immunoglobulin E
IL	interleukin
ILC2	type 2 innate lymphoid cells
MHC	myosin heavy chain
MLCK	myosin light chain kinase
MMP	matrix metalloproteinases
mRNA	messenger ribonucleic acid
NH_4_OH	ammonium hydroxide
PBS	phosphate-buffered saline
PD_20_	provocation dose of methacholine causing a 20% decrease in FEV_1_
PDGF	platelet-derived growth factor
rEOS	lung resident eosinophil
ROS	reactive oxygen species
qRT-PCR	quantitative reverse transcriptase polymerase chain reaction
SEA	severe eosinophilic asthma
SEM	standard error of the mean
SM22	transgelin
Sm	smooth muscle
TGF- *β*	transforming growth factor-beta
Th2	T helper type 2 cells
TSLP	thymic stromal lymphopoietin
VCAM-1	vascular cell adhesion molecule 1

## References

[1] Fehrenbach H., Wagner C., Wegmann M. (2017). Airway remodeling in asthma: What really matters. Cell Tissue Res..

[2] Bergeron C., Tulic M.K., Hamid Q. (2010). Airway remodelling in asthma: From benchside to clinical practice. Can. Respir. J..

[3] Ito J.T., Lourenço J.D., Righetti R.F., Tibério I., Prado C.M., Lopes F. (2019). Extracellular Matrix Component Remodeling in Respiratory Diseases: What Has Been Found in Clinical and Experimental Studies?. Cells.

[4] Araujo B.B., Dolhnikoff M., Silva L.F.F., Elliot J., Lindeman J.H.N., Ferreira D.S., Mulder A., Gomes H.A.P., Fernezlian S.M., James A. (2008). Extracellular matrix components and regulators in the airway smooth muscle in asthma. Eur. Respir. J..

[5] Hough K.P., Curtiss M.L., Blain T.J., Liu R.-M., Trevor J., Deshane J.S., Thannickal V.J. (2020). Airway Remodeling in Asthma. Front. Med..

[6] Danen E.H., Sonnenberg A. (2003). Integrins in regulation of tissue development and function. J. Pathol..

[7] Berair R., Hartley R., Mistry V., Sheshadri A., Gupta S., Singapuri A., Gonem S., Marshall R.P., Sousa A.R., Shikotra A. (2017). Associations in asthma between quantitative computed tomography and bronchial biopsy-derived airway remodelling. Eur. Respir. J..

[8] Fahy J.V. (2009). Eosinophilic and neutrophilic inflammation in asthma: Insights from clinical studies. Proc. Am. Thorac. Soc..

[9] de Groot J.C., Ten Brinke A., Bel E.H. (2015). Management of the patient with eosinophilic asthma: A new era begins. ERJ Open Res..

[10] Mesnil C., Raulier S., Paulissen G., Xiao X., Birrell M.A., Pirottin D., Janss T., Starkl P., Ramery E., Henket M. (2016). Lung-resident eosinophils represent a distinct regulatory eosinophil subset. J. Clin. Investig..

[11] Marichal T., Mesnil C., Bureau F. (2017). Homeostatic Eosinophils: Characteristics and Functions. Front. Med..

[12] Januskevicius A., Jurkeviciute E., Janulaityte I., Kalinauskaite-Zukauske V., Miliauskas S., Malakauskas K. (2020). Blood Eosinophils Subtypes and Their Survivability in Asthma Patients. Cells.

[13] Brusselle G.G., Maes T., Bracke K.R. (2013). Eosinophils in the spotlight: Eosinophilic airway inflammation in nonallergic asthma. Nat. Med..

[14] Johansson M.W., Kelly E.A., Busse W.W., Jarjour N.N., Mosher D.F. (2008). Up-regulation and activation of eosinophil integrins in blood and airway after segmental lung antigen challenge. J. Immunol..

[15] McBrien C.N., Menzies-Gow A. (2017). The Biology of Eosinophils and Their Role in Asthma. Front. Med..

[16] Halwani R., Vazquez-Tello A., Sumi Y., Pureza M.A., Bahammam A., Al-Jahdali H., Soussi-Gounni A., Mahboub B., Al-Muhsen S., Hamid Q. (2013). Eosinophils induce airway smooth muscle cell proliferation. J. Clin. Immunol..

[17] de Groot L.E.S., Sabogal Piñeros Y.S., Bal S.M., van de Pol M.A., Hamann J., Sterk P.J., Kulik W., Lutter R. (2019). Do eosinophils contribute to oxidative stress in mild asthma?. Clin. Exp. Allergy J. Br. Soc. Allergy Clin. Immunol..

[18] Salter B., Pray C., Radford K., Martin J.G., Nair P. (2017). Regulation of human airway smooth muscle cell migration and relevance to asthma. Respir. Res..

[19] Tliba O., Panettieri R.A. (2009). Noncontractile Functions of Airway Smooth Muscle Cells in Asthma. Annu. Rev. Physiol..

[20] Halwani R., Al-Muhsen S., Al-Jahdali H., Hamid Q. (2011). Role of transforming growth factor-β in airway remodeling in asthma. Am. J. Respir. Cell Mol. Biol..

[21] Janulaityte I., Januskevicius A., Kalinauskaite-Zukauske V., Palacionyte J., Malakauskas K. (2021). Asthmatic Eosinophils Promote Contractility and Migration of Airway Smooth Muscle Cells and Pulmonary Fibroblasts In Vitro. Cells.

[22] Lavinskiene S., Malakauskas K., Jeroch J., Hoppenot D., Sakalauskas R. (2015). Functional activity of peripheral blood eosinophils in allergen-induced late-phase airway inflammation in asthma patients. J. Inflamm..

[23] Cho Y.S., Moon H.B. (2010). The role of oxidative stress in the pathogenesis of asthma. Allergy Asthma Immunol. Res..

[24] Snezhkina A.V., Kudryavtseva A.V., Kardymon O.L., Savvateeva M.V., Melnikova N.V., Krasnov G.S., Dmitriev A.A. (2019). ROS Generation and Antioxidant Defense Systems in Normal and Malignant Cells. Oxidative Med. Cell. Longev..

[25] Barthel S.R., Johansson M.W., McNamee D.M., Mosher D.F. (2008). Roles of integrin activation in eosinophil function and the eosinophilic inflammation of asthma. J. Leukoc. Biol..

[26] Johansson M.W., Mosher D.F. (2013). Integrin activation States and eosinophil recruitment in asthma. Front. Pharmacol..

[27] Whetstone C.E., Ranjbar M., Omer H., Cusack R.P., Gauvreau G.M. (2022). The Role of Airway Epithelial Cell Alarmins in Asthma. Cells.

[28] Teoh C.M., Tan S.S., Tran T. (2015). Integrins as Therapeutic Targets for Respiratory Diseases. Curr. Mol. Med..

[29] Fettrelet T., Gigon L., Karaulov A., Yousefi S., Simon H.-U. (2021). The Enigma of Eosinophil Degranulation. Int. J. Mol. Sci..

[30] Gerthoffer W.T. (2008). Migration of airway smooth muscle cells. Proc. Am. Thorac. Soc..

[31] De Donatis A., Ranaldi F., Cirri P. (2010). Reciprocal control of cell proliferation and migration. Cell Commun. Signal..

[32] Ammann K.R., DeCook K.J., Li M., Slepian M.J. (2019). Migration versus proliferation as contributor to in vitro wound healing of vascular endothelial and smooth muscle cells. Exp. Cell Res..

[33] Zuyderduyn S., Sukkar M.B., Fust A., Dhaliwal S., Burgess J.K. (2008). Treating asthma means treating airway smooth muscle cells. Eur. Respir. J..

[34] Sukkar M.B., Stanley A.J., Blake A.E., Hodgkin P.D., Johnson P.R., Armour C.L., Hughes J.M. (2004). ‘Proliferative’ and ‘synthetic’ airway smooth muscle cells are overlapping populations. Immunol. Cell Biol..

[35] Parameswaran K., Radford K., Zuo J., Janssen L.J., O’Byrne P.M., Cox P.G. (2004). Extracellular matrix regulates human airway smooth muscle cell migration. Eur. Respir. J..

[36] Damera G., Tliba O., Panettieri R.A. (2009). Airway smooth muscle as an immunomodulatory cell. Pulm. Pharmacol. Ther..

[37] Verrecchia F., Mauviel A. (2002). Transforming growth factor-beta signaling through the Smad pathway: Role in extracellular matrix gene expression and regulation. J. Investig. Dermatol..

[38] Ojiaku C.A., Yoo E.J., Panettieri R.A. (2017). Transforming Growth Factor β1 Function in Airway Remodeling and Hyperresponsiveness. The Missing Link?. Am. J. Respir. Cell Mol. Biol..

[39] Ohno I., Nitta Y., Yamauchi K., Hoshi H., Honma M., Woolley K., O’Byrne P., Tamura G., Jordana M., Shirato K. (1996). Transforming growth factor beta 1 (TGF beta 1) gene expression by eosinophils in asthmatic airway inflammation. Am. J. Respir. Cell Mol. Biol..

[40] Ito I., Fixman E.D., Asai K., Yoshida M., Gounni A.S., Martin J.G., Hamid Q. (2009). Platelet-derived growth factor and transforming growth factor-beta modulate the expression of matrix metalloproteinases and migratory function of human airway smooth muscle cells. Clin. Exp. Allergy J. Br. Soc. Allergy Clin. Immunol..

[41] Fukushima T., Yamasaki A., Harada T., Chikumi H., Watanabe M., Okazaki R., Takata M., Hasegawa Y., Kurai J., Yanai M. (2017). γ-Tocotrienol Inhibits TGF-β1-Induced Contractile Phenotype Expression of Human Airway Smooth Muscle Cells. Yonago Acta Med..

[42] Hinz B. (2015). The extracellular matrix and transforming growth factor-β1: Tale of a strained relationship. Matrix Biol..

[43] Ohno I., Ohtani H., Nitta Y., Suzuki J., Hoshi H., Honma M., Isoyama S., Tanno Y., Tamura G., Yamauchi K. (1997). Eosinophils as a source of matrix metalloproteinase-9 in asthmatic airway inflammation. Am. J. Respir. Cell Mol. Biol..

[44] Han S.W., Roman J. (2006). Fibronectin induces cell proliferation and inhibits apoptosis in human bronchial epithelial cells: Pro-oncogenic effects mediated by PI3-kinase and NF-kappa B. Oncogene.

[45] Duronio R.J., Xiong Y. (2013). Signaling pathways that control cell proliferation. Cold Spring Harb. Perspect. Biol..

[46] Al-Muhsen S., Johnson J.R., Hamid Q. (2011). Remodeling in asthma. J. Allergy Clin. Immunol..

[47] Samitas K., Carter A., Kariyawasam H.H., Xanthou G. (2018). Upper and lower airway remodelling mechanisms in asthma, allergic rhinitis and chronic rhinosinusitis: The one airway concept revisited. Allergy.

[48] Redza-Dutordoir M., Averill-Bates D.A. (2016). Activation of apoptosis signalling pathways by reactive oxygen species. Biochim. Biophys. Acta.

[49] Hellewell A.L., Rosini S., Adams J.C. (2017). A Rapid, Scalable Method for the Isolation, Functional Study, and Analysis of Cell-derived Extracellular Matrix. J. Vis. Exp. JoVE.

[50] Scherzer M.T., Waigel S., Donninger H., Arumugam V., Zacharias W., Clark G., Siskind L.J., Soucy P., Beverly L. (2015). Fibroblast-Derived Extracellular Matrices: An Alternative Cell Culture System That Increases Metastatic Cellular Properties. PLoS ONE.

[51] Chen Y., Chen J., Zhang Z., Lou K., Zhang Q., Wang S., Ni J., Liu W., Fan S., Lin X. (2017). Current advances in the development of natural meniscus scaffolds: Innovative approaches to decellularization and recellularization. Cell Tissue Res..

[52] Mathur S.K., Schwantes E.A., Jarjour N.N., Busse W.W. (2008). Age-related changes in eosinophil function in human subjects. Chest.

[53] Koussounadis A., Langdon S.P., Um I.H., Harrison D.J., Smith V.A. (2015). Relationship between differentially expressed mRNA and mRNA-protein correlations in a xenograft model system. Sci. Rep..

[54] Gosens R., Stelmack G.L., Dueck G., McNeill K.D., Yamasaki A., Gerthoffer W.T., Unruh H., Gounni A.S., Zaagsma J., Halayko A.J. (2006). Role of caveolin-1 in p42/p44 MAP kinase activation and proliferation of human airway smooth muscle. Am. J. Physiol. Lung Cell. Mol. Physiol..

[55] Liang C.C., Park A.Y., Guan J.L. (2007). In vitro scratch assay: A convenient and inexpensive method for analysis of cell migration in vitro. Nat. Protoc..

[56] Crow J.P. (1997). Dichlorodihydrofluorescein and dihydrorhodamine 123 are sensitive indicators of peroxynitrite in vitro: Implications for intracellular measurement of reactive nitrogen and oxygen species. Nitric Oxide Biol. Chem..

[57] Djiadeu P., Azzouz D., Khan M.A., Kotra L.P., Sweezey N., Palaniyar N. (2017). Ultraviolet irradiation increases green fluorescence of dihydrorhodamine (DHR) 123: False-positive results for reactive oxygen species generation. Pharmacol. Res. Perspect..

[58] Balaiya S., Chalam K.V. (2014). An In vitro Assay to Quantify Nitrosative Component of Oxidative Stress. J. Mol. Genet. Med. Int. J. Biomed. Res..

